# A cut-off of 2150 cytokeratin 19 mRNA copy number in sentinel lymph node may be a powerful predictor of non-sentinel lymph node status in breast cancer patients

**DOI:** 10.1371/journal.pone.0171517

**Published:** 2017-02-10

**Authors:** Irene Terrenato, Valerio D’Alicandro, Beatrice Casini, Letizia Perracchio, Francesca Rollo, Laura De Salvo, Simona Di Filippo, Franco Di Filippo, Edoardo Pescarmona, Marcello Maugeri-Saccà, Marcella Mottolese, Simonetta Buglioni

**Affiliations:** 1 Biostatistics Unit, Regina Elena National Cancer Institute, Rome, Italy; 2 Department of Pathology, Regina Elena National Cancer Institute, Rome, Italy; 3 Department of Surgery, Regina Elena National Cancer Institute, Rome, Italy; 4 Division of Medical Oncology B, Regina Elena National Cancer Institute, Rome, Italy; 5 Scientific Direction, Regina Elena National Cancer Institute, Rome, Italy; University of Toronto, CANADA

## Abstract

Since 2007, one-step nucleic acid amplification (OSNA) has been used as a diagnostic system for sentinel lymph node (SLN) examination in patients with breast cancer. This study aimed to define a new clinical cut-off of CK19 mRNA copy number based on the calculation of the risk that an axillary lymph node dissection (ALND) will be positive. We analyzed 1529 SLNs from 1140 patients with the OSNA assay and 318 patients with positive SLNs for micrometastasis (250 copies) and macrometastasis (5000 copies) underwent ALND. Axillary non–SLNs were routinely examined. ROC curves and Youden’s index were performed in order to identify a new cut-off value. Logistic regression models were performed in order to compare OSNA categorical variables created on the basis of our and traditional cut-off to better identify patients who really need an axillary dissection. 69% and 31% of OSNA positive patients had a negative and positive ALND, respectively. ROC analysis identified a cut-off of 2150 CK19 mRNA copies with 95% sensitivity and 51% specificity. Positive and negative predictive values of this new cut-off were 47% and 96%, respectively. Logistic regression models indicated that the cut-off of 2150 copies better discriminates patients with node negative or positive in comparison with the conventional OSNA cut-off (p<0.0001). This cut-off identifies false positive and false negative cases and true-positive and true negative cases very efficiently, and therefore better identifies which patients really need an ALND and which patients can avoid one. This is why we suggest that the negative cut-off should be raised from 250 to 2150. Furthermore, we propose that for patients with a copy number that ranges between 2150 and 5000, there should be a multidisciplinary discussion concerning the clinical and bio-morphological features of primary breast cancer before any decision is taken on whether to perform an ALND or not.

## Introduction

Sentinel lymph node (SLN) biopsy is currently the recommended procedure for axillary staging in clinically node-negative early breast cancer at diagnosis. When patients are positive for SLN, complete ALND is usually performed but the non-sentinel lymph nodes (non–SLN) of 40%-70% of these patients are found not to have metastases [1–3]. This is one of the reasons why the role of ALND in the surgical management of breast cancer patients with a positive SLN has changed considerably in recent years.

The American College of Surgeons Oncology Group (ACOSOG) Z0011 trial has defined a select cohort of patients with positive SLN in which a complete ALND may be safely avoided [4]. However, there is still a number of patients where the prediction of non-SLN metastasis may be helpful when deciding whether or not to perform an ALND.

Multiple studies have aimed to identify predictive variables of non-SLN metastases in order to select those patients who can be spared complete ALND. To this end, many nomograms [5, 6] have been proposed but all of these show some inconsistencies. First of all, many of them are based on histological information obtained by means of post-operative histological examination of SLN and tumor tissue. Furthermore, most of the variables considered in these studies are subjective and difficult to reproduce.

Since 2007, one-step nucleic acid amplification (OSNA) has been used as a diagnostic semi-automatic system for sentinel lymph node examination in patients with breast cancer [7–16]. The OSNA assay is based on a rapid real-time amplification and quantification of Cytokeratin 19 mRNA copy numbers in homogenized samples of lymph nodes. Tsujimoto and colleagues [17], who first described OSNA, used the mRNA copy number as a surrogate for metastatic sentinel lymph node positivity. Cut-off values were defined in order to discriminate negative nodes from micrometastases and macrometastases. They set the cut-off value at 2.5 x10^2^ CK19 mRNA copies/μL, which represents the upper limit of the copy numbers in the histopathologically negative lymph nodes from pN0 patients. To obtain a cut-off value for CK19 mRNA expression between micrometastases (250–5000 CK19 mRNA copies/μL) and macrometastases (more than 5000 CK19 mRNA copies/μL) they correlated the volume of metastatic foci of histopathologically positive lymph nodes with CK 19 mRNA expression.

To date, more than 100,000 SLNs in nearly 200 European hospitals have been analyzed applying these cut-off values in clinical practice and more than 80 studies have been published demonstrating the reliability of the molecular OSNA assay in routine clinical breast cancer therapy.

Among these studies, our previous paper [18] demonstrated that a specific cut-off of 2000 CK19 mRNA copy numbers in the SLN could predict the likelihood of finding positive axillary lymph nodes. This copy number was obtained from the molecular analysis of only half of the SLNs based on a 4-slice mode and taking into account breast cancer molecular classification. In order to confirm the cut-off value established in our previous study, the present investigation was conducted on a new prospective and consecutive series of early BC patients treated in our Institute for SLN biopsy. The current study is based on a large sample (the biggest in Europe) in which the whole SLN is analyzed by the molecular OSNA method.

We propose to verify and then use the new cut-off value established according to risk and not according to the traditional method that only considers the dimensions of the metastases. In this way, the clinical use of OSNA, based on objective and reproducible data, can be further optimized by considering a risk-based cut-off value. With this approach, therefore, the histological concept of micrometastasis and macrometastasis could be overcome in favor of an objective evaluation of CK19 mRNA copy number.

## Materials and methods

### Study population

The ethics committee of the Regina Elena National Cancer Institute (Prot. CE/913/10). reviewed and approved this study. In our prospective study, we included 1529 fresh SLNs sampled from 1140 consecutive patients with a tumor with a maximum diameter of 5 cm or less and with clinically negative axillary lymph nodes.

This research was carried out between January 2011 and June 2014. We excluded from the study BC patients presenting T3 –T4 tumors, patients already treated for a BC, patients submitted to neoadjuvant therapy, or previously affected by a non BC tumor.

In all the patients, ER, PgR, HER2 and Ki-67 were evaluated on a core biopsy of the primary tumor before breast surgery and SLN biopsy.

Patients were subjected to modified radical mastectomy or quadrantectomy and, in cases where the OSNA assay was positive, ALND was performed during the same surgical session of the SLN biopsy. Tumors were graded according to Bloom and Richardson and staged according to the Unione Internationale Contre le Cancer tumor-node-metastasis (TNM) system criteria [19].

A written informed consent was obtained from all patients before surgical procedures. In order to define a specific cut-off of CK19 mRNA copy number, we focused our study on the 318 patients with positive sentinel lymph node who underwent axillary dissection. Table 1 summarizes the clinical-pathological characteristics of the 318 patients included in our study.

**Table 1:** Clinico-pathological characteristics of the patients (N = 318).

Characteristics		N (%)
	Invasive ductal carcinoma	286 (90)
	Invasive lobular carcinoma	30 (9)
	Other carcinomas	2 (1)
**Histological grade**		
	Grade 1- Grade 2	241 (76)
	Grade 3	77 (24)
**Tumor size**		
	T1	207 (65)
	T2	111 (35)
**LVI**		
	Negative	135 (43)
	Positive	183 (57)

### Intraoperative SLN analysis with the OSNA assay

SLN biopsy was performed using technetium 99m-labeled, nano-sized, human serum albumin colloids. The fat tissue was removed from the harvested, fresh sentinel lymph nodes, which were then weighted and measured. The mean number of SLNs was 1.2 per patient. SLNs weighing less than 50 mg were excluded from the study. SLNs weighing more than 600 mg were further subdivided into two samples and separate analyses were carried out. Whole SLNs were examined by OSNA method with the gene amplification detector RD100i (Sysmex, Kobe, Japan) according to the manufacturer’s instructions. Briefly, lymph nodes were homogenized with 4 ml of a lysis buffer (Sysmex) on ice and after centrifugation the middle phase, containing the target mRNA, was separated from the homogenate and subjected to RT-LAMP reaction of CK19 mRNA while the excess lysate was stored at −80°C.

According to the manufacturer instructions, the cut-off values for OSNA macrometastases (++) were >5000 copies/μl of CK19 mRNA, while for micrometastases (+), the value was from 250 to 5000 copies/μl. SLNs were considered negative when the CK19 mRNA results were <250 copies/μl

When more than one SLN was positive, we considered the total tumor load as the sum of the CK19 mRNA copy numbers. The OSNA results were immediately communicated by telephone to the Surgery Department within 30–40 minutes. ALND was performed both on patients with micrometastases (+) and macrometastases (++), whereas in patients with an OSNA negative result, no further axillary surgery was done. Axillary non-SLNs were routinely examined by H&E staining and the number of positive lymph nodes was recorded.

### Immunohistochemistry

Immunoreactions were revealed by Bond Polymer Refine Detection on an automated autostainer (Bond^™^ Max, Leica Biosystem, Milan, Italy). ER and PgR expression was evaluated using mAb 6F11 (Leica, Novocastra) and mAb 1A6 (Leica, Novocastra) respectively. Cell proliferation and HER2 overexpression were tested using the Ki-67 mAb (MIB1, Dako, Milan, Italy) and the polyclonal antibody A0485 (Dako), respectively. Tumors were also characterized for cytokeratin 19 expression (Dako). HER2 IHC positivity was defined according to ASCO/CAP guidelines [20]. ER and PgR were considered positive when >1% and ≥20% of the neoplastic cells showed distinct nuclear immunoreactivity, respectively. Ki-67, based on the median value of our series, was regarded as high if more than 15% of the cell nuclei were immunostained. Evaluation of the IHC results, blinded to all patient data, was performed independently and in blinded manner by two investigators (SB and MM).

### Silver in situ hybridization

HER2 gene amplification was evaluated using a fully automated dual color in situ hybridization DNA probe assay (DDISH, Ventana, Roche Diagnostics, Milan, Italy). The assay was performed according to the manufacturer's instructions. DDISH results were analyzed by using a light microscope (Nikon, Eclipse 55i, Moncalieri (To) Italy) equipped with a software able to capture images (Eureka Interface System, Menarini, Florence, Italy). HER2 was defined amplified by DDISH when a HER2 gene copy number ≥6 or a ratio ≥2 was detected in at least 60 nuclei in 6 to 8 randomly selected invasive tumor areas. Lymphocytes and normal breast glandular epithelial cells served as internal controls.

### Statistical analysis

Descriptive statistics were calculated for all variables of interest. Categorical variables were reported as frequencies and percentage values, while continuous variables were plotted into box-plots. The method of analysis and the tests for statistical significance depended on the variables under study.

Receiver operative characteristics (ROC) curves were used in order to search for an optimal cut-off value with the highest sensitivity and specificity and Youden’s index was performed in order to identify a new cut-off value.

Across various cut-off points, Youden’s index maximized the difference between sensitivity and specificity and between real-positive and false-positive subjects. Thus, the optimal cut-off value was calculated.

Sensitivity (Se), specificity (Sp), negative and positive predictive value (NPV and PPV) with their relative 95% confidence interval (CI) of the OSNA assay classified according to the new cut-off were estimated considering ALND as the gold standard.

Logistic regression models were used to estimate crude and adjusted odd ratios associated with positive ALND. Multivariate models were run including variables that were found to be significant after univariate analysis. P-values <0.05 were considered statistically significant. The SPSS (version 21.0) statistical program (SPSS Inc., Chicago, IL, USA) was used for our analyses.

## Results

### OSNA assay results and management of the axilla

In our sample of 1140 early breast cancer patients, 318 (27.8%) had a positive SLN. Taking into account the entire series of patients included in our study, the percentage of cases with a positive SLN for micrometastases (OSNA+, 250–5000 copies) was 12.8% (146/1140), whereas the percentage for macrometastases (OSNA++) was 15% (172/1140). Of the 318 patients with a positive OSNA assay, 220 (69%) had a negative ALND and 98 (31%) were positive. Interestingly, of the 220 negative ALND, 125 (57%) were OSNA+ patients and 95 (43%) were OSNA++. In contrast, of the 98 positive ALND, 77 (79%) had macrometastases in the SLN and only 21 (21%) had micrometastases (Table 2).

**Table 2:** Relationship between ALND and SLN status according to traditional cut-off (N = 318).

		ALND positive	ALND negative	
		N (%)	N (%)	
OSNA assay				Total
	**Macrometastasis** (>5000 copies)	77 (79%)	95 (43%)	172
	**Micrometastasis** (250–5000 copies)	21 (21%)	125 (57%)	146
	**Total**	98 (100%)	220 (100%)	318

### Biological characteristics of the 318 BC patients with a positive SLN

In order to study the OSNA results within the five different subtypes, we stratified our series of 318 invasive BC patients with positive sentinel lymph node by molecular classification according to the surrogate definitions of intrinsic subtypes of breast cancer modified during the last St Gallen Conference [21]: “Luminal A-like” (all of: ER and PgR positive, HER2 negative and Ki-67 low); “Luminal B-like HER2 negative” (ER positive, HER2 negative and at least one of: Ki-67 high, PgR negative or low); “Luminal B-like HER2 positive” (ER positive, HER2 over-expressed or amplified, any Ki-67, any PgR); “HER2 positive non-luminal” (HER2 over-expressed or amplified, ER and PgR absent); “Triple negative ductal” (ER and PgR absent, HER2 negative). As shown in Table 3, we found that 141 BC (44%) were LA, 103 (32%) were LBH-, 44 (14%) were LBH+, 14 (5%) were HS, and 16 (5%) were TN.

**Table 3:** Biological findings of 318 patients with positive sentinel lymph-node who underwent axillary dissection (N = 318).

Characteristics		N (%)
**Age in years**	(Median, min-max)	**54 (31–81)**
Subtypes*		
	Luminal A	141 (44)
	Luminal B H-	103 (32)
	Luminal B H+	44 (14)
	HER2 Subtype	14 (5)
	Triple Negative	16 (5)

*LA (ER+/PgR+, HER2-, Ki-67 low); LB H- (ER+, HER2-, PgR- and/or Ki-67 high); LB H+ (ER+ HER2+, PgR- and/or Ki-67 high); HS (ER-,PgR-, HER2+,any Ki-67); TN (ER-,PgR-, HER2- any Ki-67).

### Correlation between CK19 mRNA copy number in positive SLN and the risk of axillary involvement

Results from ROC analyses, with an AUC of 0.765, identified a cut-off equal to 2150 CK19 mRNA copies. Therefore, two groups were identified. The first group included 118 patients having ≤2150 of CK19 mRNA copies. Of these, 113 (96%) were ALN-negative and 5 (4%) ALN-positive, with one or more positive lymph nodes (false negative cases). Interestingly, none of the five cases was a Luminal A-like subtype and all of them had a high Ki-67. They were also characterized by a tumor size >1 cm. The second group included 200 patients with more than 2150 CK19 mRNA copies; 93 (46%) ALN-positive, with one or more positive lymph nodes (true positive cases) and 107 (54%) were ALN-negative (false positive cases). Within this group, we observed that patients who were ALN-negative showed better prognostic characteristics than ALN-positive. In particular, they were mostly luminal with negative HER2 (p = 0.023).

Interestingly if we had used our cut-off of 2150 copies instead of 250, the results would have been very different and we could have avoided an unnecessary ALND in a significant number of patients.

**Fig 1:**
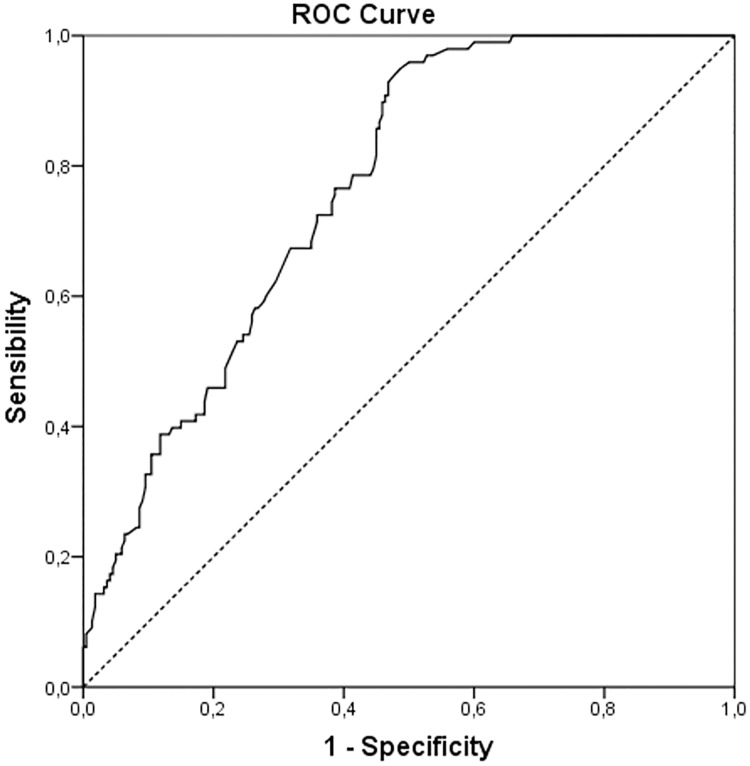
ROC curve. The new cut-off of cytokeratin 19 mRNA copy number obtained by the ROC curve. Youden’s index identifies the optimal value at 2150 copies.

The new cut-off showed 94.9% sensitivity and 51.4% specificity when differentiating patients with negative ALN or with one or more metastatic lymph nodes (Fig 1). Positive and negative predictive values of this new cut-off were 46.5% and 95.8% respectively.

Table 4 shows the diagnostic performance of the new cut-off in comparison with the conventional and the two recently proposed cut-off [22–23]. Our threshold demonstrates a higher sensitivity and negative predictive value in differentiating patients with positive ALND.

**Table 4:** Comparison of our new proposed OSNA cut-off with the conventional figure and two other alternatives from literature in the identification of patients with positive ALND.

	Newly proposed cut-off*	Conventional cut-off**	De Ambrogio cut-off***	Peg cut-off****
**Sensibility**	94.9%	78.6%	72.4%	62.2%
**Specificity**	51.4%	56.8%	61.8%	70.5%
**PPV**	46.5%	44.8%	45.8%	48.4%
**NPV**	95.8%	85.6%	83.4%	80.7%
**FN**° **cases (N, %)**	5 (1.6%)	21 (6.6%)	27 (8.5%)	37 (11.6%)
**FP**°° **cases (N, %)**	107 (33.6%)	95 (29.9%)	84 (26.4%)	65 (20.4%)

*2150 copies;

** 5000 copies;

***7700 copies;

**** 15000 copies

° FN:False Negative;

°° FP: False Positive

In addition, we evaluated the correlation between the CK19 mRNA copy number and the number of positive non-sentinel lymph nodes. We obtained a Pearson’s correlation coefficient equal to 0.484 (p<0.0001), which shows a moderate, direct relationship between these two parameters.

### Relationship between SLN copy number and molecular subtypes

**Fig 2:**
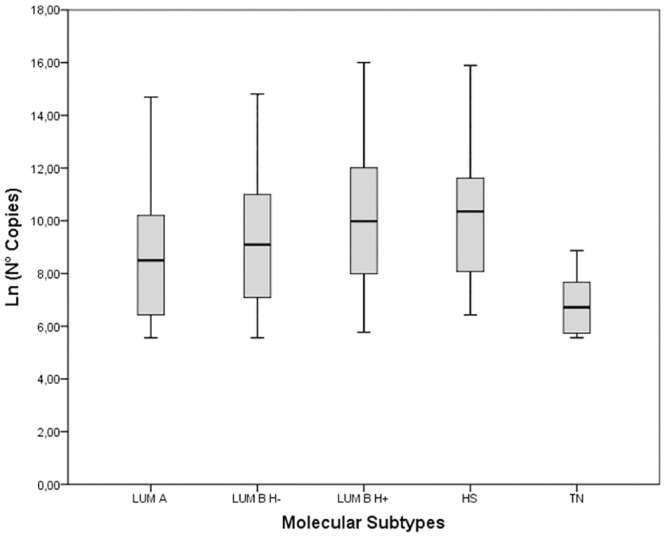
Cytokeratin 19 mRNA copy number distribution according to molecular subtypes. Box Plot showing the distribution of cytokeratin 19 mRNA copy number in the five molecular subtypes. The Kruskall-Wallis test indicates a significant difference among groups (p<0.0001). Data are expressed on a log scale for presentation purposes.

Through the Kruskall-Wallis test, we quantified the average copy number distribution of 5 molecular subtypes. As we can clearly see from the graph (Fig 2), the average values increase constantly and significantly (p value <0.0001) from the LA subtype (mean 119,203) to the HS subtype (mean 727,899). However, TN subtype has the lowest average copy number because it was characterized by a high number of patients with negative or micrometastatic SLNs.

### Logistic regression analysis

We carried out univariate and multivariate logistic regression models taking into account the following variables: BC subtypes, LVI, tumor grading, tumor size, OSNA assay with the traditional cut-off and with the cut-off obtained by ROC curve. In particular, the multivariate model (Table 5) indicates that the probability of an ALND negative is significantly higher in patients with LA subtype tumor, negative LVI, SLN OSNA+ and ≤2150 copies. Furthermore, these findings indicated that the cut-off of 2150 copies better detects patients with negative ALND (OR 19.6, 95% CI 7.7–50.2; p<0.0001) in comparison with the conventional OSNA cut-off of 5000 copies (OR 4.83, 95% CI 2.8–8.4; p<0.0001).

**Table 5:** Logistic regression analysis of variables that might predict non-sentinel node involvement in 318 patients with metastatic SLNs. Univariate and Multivariate analysis.

		Probability of a negative ALND					
		Univariate Model			Multivariate Model		
		OR	95% CI	p-value	OR	95% CI	p-value
Subtypes*	**LA vs LB H-**	**1.81**	**1.02–3.26**	**0.043**	1.53	0.77–3.03	0.223
	**LA vs LB H+**	**4.63**	**2.26–9.53**	**<0.0001**	**2.93**	**1.23–6.99**	**0.015**
	**LA vs HS**	**5.15**	**1.66–16.01**	**0.005**	2.45	0.66–9.11	0.183
	**LA vs TN**	1.29	0.39–4.29	0.681	**5.30**	**1.12–25.14**	**0.036**
LVI**	**Negative vs Positive**	**6.04**	**3.32–10.98**	**<0.0001**	**3.91**	**1.99–7.69**	**<0.0001**
**Grading**	**G1+G2 vs G3**	**2.73**	**1.60–4.65**	**<0.0001**	1.49	0.78–2.84	0.226
**Tumor size**	**T1 vs T2**	**2.40**	**1.45–3.96**	**0.001**	1.35	0.74–2.45	0.331
**OSNA assay**	**≤5000 vs >5000**	**4.83**	**2.78–8.37**	**<0.0001**			
	**≤2150 vs >2150**	**19.64**	**7.69–50.18**	**<0.0001**	**17.71**	**6.16–50.94**	**<0.0001**

*LA: Luminal A; LB H-: Luminal B HER2-; LB H+: Luminal B HER2+; HS: HER2 subtype; TN: Triple Negative.

**LVI: Lymphovascular Invasion.

## Discussion

An increasing number of published articles in recent years has demonstrated the consistency of the OSNA assay in detecting metastases in the SLN.

Tsujimoto and colleagues [17], who first described OSNA, established the OSNA cut- off values to discriminate negative SLN, from micrometastatic and macrometastatic SLN, using criteria derived from histology. To date, all the OSNA users have adopted these cut-off values when deciding on whether to perform an axillary dissection.

In this study, we tried to overcome the traditional histological distinction between micrometastases and macrometastases so we considered the overall tumor load by analysing the risk of positive ALN in correlation with CK19 mRNA copy number in SLN following detection by OSNA.

We constructed a ROC curve to establish the best cut-off value in terms of sensitivity and specificity. We found that a cut-off of >2150 CK19 mRNA copies identifies false and true positive and negative cases very efficiently, and therefore it better identifies which patients really need an ALND and which patients do not.

In an our previous study [18], in which we used only 2 alternate slices of each sentinel lymph node, we focused on OSNA+ BC patients (micrometastatic SLN) and we observed that only cases with negative ALND presented a CK19 mRNA copy number <2000. Conversely, all the cases with positive ALND had a CK19 mRNA copy number >2000. In this current study, however, we included a new consecutive, prospective and much larger series of BC patients analyzing not just part, but the entire SLN. We found a cut-off value which was practically identical to the figure we had established in our earlier study. This new data confirm and validate our previously published data.

Other studies have tried to define a specific cut-off of CK19 mRNA copy number.

Peg et al [23] proposed a total tumor load of 15,000 copies as reliable predictor of axillary status in early breast cancer. Specifically, using the above cut-off, they reported a NPV of 85.5%, a PPV of 41.1%, a sensitivity of 76.7% and a specificity of 55.2%. These values, however, compare unfavorably to those we obtained in our series upon applying the 2150 copies cut-off value (NPV 95.8% NPV, 46.5% PPV, 94.9% sensitivity and 51.4% specificity). In addition, by directly comparing the 2150 and 15,000 copies cut-off values in our own series, we clearly demonstrated the superiority of the 2150 copies cut-off in assessing axillary node status.

Deambrogio et al. [22] demonstrated that a specific cut-off of 7700 copies may be useful to identify patients with one or more positive non-SLN, independently of the characteristics of the primary tumor. However, in Deambrogio’s work, only 60% of their breast cancer patients with a positive SLN for micrometastasis underwent an axillary dissection. The remaining 40% had no further surgical treatment. Naturally, this choice strongly influenced the results of the ROC analysis.

Heilmann et al. [24] found that a cut-off CK19 mRNA copy number of 7900 obtained using ROC analysis indicates a positive non-SLN result with the highest sensitivity and specificity. This result was obtained by separately processing 1 mm central slides for histology and the rest for OSNA, and therefore it might not represent the real copy number of CK19 mRNA of the entire lymph node. Moreover, this cut-off was obtained from a very small cohort of 143 patients of which only 39 had a positive SLN.

On the contrary, in our study, we considered all the SLN with a copy number above 250 to be positive and all these patients underwent an axillary lymph node dissection. Thanks to this surgical approach, today, we have a complete and global dataset regarding the axillary status of a large and consecutive cohort of breast cancer patients.

In our opinion, the value of CK19 mRNA 7700/7900 copies is too high and could prevent the identification of BC patients with a positive ALND. In our current series, in fact, 23 out of the 50 cases (46%) with a CK19 mRNA copy number between 2150 and 7700 had a positive ALND (data not shown). Furthermore, it is important to underline that the cut-off value of >2150 copies was identified in two different and particularly large series of BC patients, the first of 709 patients and the current of 1140 patients for a total of 1849 patients.

Although our cut-off of 2150 CK 19 copy number could be used independently of the characteristics of the primary tumor, we delved deeper into the matter and we examined the likelihood of a positive or negative ALND according to the new cut-off value taking concomitantly into account the different molecularly distinct BC subtypes.

In order to better understand the clinical implications of the new cut-off, we studied the main bio-pathological features of the false negative and false positive patients and we observed a strict correlation between the molecular subtypes and the real risk of having a positive ALND. Interestingly, none of the false negative patients was a Luminal A subtype and all of them had a high Ki-67, while the subset of false positive patients showed a significantly higher percentage of Luminal A subtype compared to the true positive one.

In the light of these results, we evaluated the relationship between SLN copy number and molecular subtypes through the Kruskal-Wallis (KW) statistical test.

The KW test showed that the average values of copy number increase constantly and significantly from the LA subtype to the HS subtype. Interestingly, the TN tumors showed the lowest average copy number and the lowest risk of having high-volume nodal disease. This observation is consistent with several published series that relate these characteristics to molecular subtypes. Recently, Ugras et al. [25] demonstrated in a very large retrospective analysis that TN tumors have the lowest risk of any subtype of having LN metastases, despite the uniformly worse prognosis and increased rate of LR in these tumors.

All these findings were statistically supported by univariate and multivariate analysis. In fact, the model indicated that the probability of finding an ALND positive was significantly higher in patients with LBH+, HS, LVI positive, OSNA++ and OSNA >2150 copies. Our results, in line with Peg et al. [23], demonstrated that the OSNA assay significantly predicts ALND positivity mainly when associated with unfavorable bio-pathological parameters. Moreover, logistic regression indicated that the cut-off of >2150 copies better discriminated patients with positive or negative ALND in comparison with the conventional OSNA cut-off of 5000 copies.

In our Institute in more than 90% of the early breast cancer patients, ER, PgR, HER2 and Ki-67 are evaluated on a core biopsy of the primary tumor before breast surgery and SLN biopsy. For these reasons, we can establish the biological profile of the primary tumor before the sentinel biopsy. This approach is particularly important for patients with a SLN with an intermediate copy number (2150–5000) which represented 5% of the entire series of early breast cancer patients.

The current NCCN guidelines clearly recommend that sentinel lymph node-negative breast cancer patients do not require ALND, whereas the effect of ALND in SLN-positive early breast cancer remains controversial. The meta analysis by Li et al. [26] which systematically reviewed the current studies regarding the safety and efficacy of SLNB alone versus ALND in early breast cancer with SLN metastasis, highlights that high-quality prospective trials on larger populations are needed to further clarify this controversial issue.

However, a positive SLN, which reflects the nodal tumor burden, is of particular clinical value as a predictive tool for further non-sentinel involvement. In this context, the importance of a standardized OSNA cut-off, is even more relevant, not only for surgical decision, but also for staging and therapy.

Another effective approach to assess the risk of positive ALND could be the incorporation of the results of the OSNA assay into a nomogram. Two large multicentric studies, which developed nomograms to predict non-SLN positivity based on the CK19 mRNA copy number determined by OSNA assay, have been recently published [27, 28]. Nevertheless, the two proposed nomograms do not appear to be of particular clinical usefulness. In fact, in both studies, the variables statistically associated with OSNA assay are tumor size and LVI, parameters that can be accurately detected only after surgery of the primary tumor.

In conclusion, considering literature and available data, what is certain is that the ALND can be excluded in SLN negative patients. Thus, it is important to establish the ideal negative cut-off which best identifies true negative patients that do not need an ALND.

To this end, we suggest that the negative cut-off of OSNA assay should be raised from 250 to 2150. Furthermore, we propose that, for patients with a copy number that ranges between 2150 and 5000, the decision on whether to perform an ALND or not should be taken after a multidisciplinary discussion concerning the clinical and bio-morphological features of primary breast cancer.

In order to avoid an under treatment of breast cancer patients with a positive SLN, a multidisciplinary team approach, and excellent communication and cooperation between clinicians is essential to ensure that each patient’s outcome is maximized while minimizing morbidity.

## Supporting information

S1 Data Concerns the clinical information of the 318 breast cancer patients included in our study. (XLSX)
